# Probing the Kinetic and Thermodynamic Fingerprints of Anti-EGF Nanobodies by Surface Plasmon Resonance

**DOI:** 10.3390/ph13060134

**Published:** 2020-06-26

**Authors:** Salvador Guardiola, Monica Varese, Marta Taulés, Mireia Díaz-Lobo, Jesús García, Ernest Giralt

**Affiliations:** 1 Institute for Research in Biomedicine (IRB Barcelona), The Barcelona Institute of Science and Technology (BIST), 08028 Barcelona, Spain; salvador.guardiola@irbbarcelona.org (S.G.); monica.varese@irbbarcelona.org (M.V.); jesus.garcia@irbbarcelona.org (J.G.); 2 Scientific and Technological Centres (CCiT-UB), Molecular Interactions, University of Barcelona, 08028 Barcelona, Spain; marta@ccit.ub.edu; 3 Mass Spectrometry and Proteomics Core Facility, Institute for Research in Biomedicine (IRB Barcelona), The Barcelona Institute of Science and Technology (BIST), 08028 Barcelona, Spain; mireia.diaz@irbbarcelona.org; 4 Department of Inorganic and Organic Chemistry, University of Barcelona, 08028 Barcelona, Spain

**Keywords:** nanobodies, SPR, biophysics, thermodynamics, molecular recognition, EGF, antibody–antigen (Ag–Ab) interactions

## Abstract

Despite the widespread use of antibodies in clinical applications, the precise molecular mechanisms underlying antibody–antigen (Ab–Ag) interactions are often poorly understood. In this study, we exploit the technical features of a typical surface plasmon resonance (SPR) biosensor to dissect the kinetic and thermodynamic components that govern the binding of single-domain Ab or nanobodies to their target antigen, epidermal growth factor (EGF), a key oncogenic protein that is involved in tumour progression. By carefully tuning the experimental conditions and transforming the kinetic data into equilibrium constants, we reveal the complete picture of binding thermodynamics, including the energetics of the complex-formation transition state. This approach, performed using an experimentally simple and high-throughput setup, is expected to facilitate mechanistic studies of Ab-based therapies and, importantly, promote the rational development of new biological drugs with suitable properties.

## 1. Introduction

Antibody–antigen (Ab–Ag) interactions are one of the most relevant classes of protein-mediated molecular recognition processes. They have been selected during millions of years of evolution for their ability to bind with high specificity and affinity to a wide variety of antigenic molecules [1,2]. From a pharmaceutical perspective, the last decade has witnessed an explosion in the use of Ab for imaging [3], drug conjugation [4], diagnostic [5] and therapeutic [6] purposes, among many others. Although the key strengths of Abs are widely recognised in terms of affinity, specificity and biochemical stability, full-length monoclonal antibodies (mAbs) suffer from some limitations when it comes to their clinical development, such as undesired Fc-mediated cytotoxicity, poor penetration in target tissues, aggregation and stability problems, low batch-to-batch reproducibility, and elevated manufacturing costs, among others [7]. To overcome these issues, a variety of Ab formats have been developed and optimised in recent years, thanks to the remarkable progress achieved in protein engineering and in vitro display methods [8,9]. Among these Ab formats, camelid-derived single-domain Abs, also known as VHHs or nanobodies, are the smallest Ag-binding proteins found in nature and have attracted great interest for biotechnological and pharmaceutical applications [10]. Unlike conventional Abs, nanobodies are formed by a single chain of only ~14 kDa and they can be readily expressed in recombinant bacteria with high yields [11]. Despite their small size, being one-tenth of a standard IgG antibody, nanobodies can engage their targets with similar affinities and specificity to full-length Abs. In addition, compared to mAb, they have a longer variable CDR3 loop, which forms part of a bulging interface that is ideally suited for targeting cavities, grooves and flexible epitopes [12].

Despite the undeniable progress achieved with the development of therapeutic Abs, there are relatively few studies shedding light on the kinetics and thermodynamics that drive antigen recognition [13,14,15]. Moreover, some of these studies have been performed with model systems consisting of full-length Abs that recognise small-molecule antigens [16,17]. On the contrary, the analysis of pharmacologically relevant Ab–Ag interactions, at the mechanistic level, is of utmost importance for the success of therapeutic antibodies during preclinical stages of development.

Protein–protein interactions in general, and antibody–antigen interactions in particular, can be characterised at several quantitative levels. One of the simplest descriptors is defined by the dissociation constant (*K*_D_), a measure of the strength of the interaction at equilibrium. The *K*_D_ may be further resolved into its kinetic descriptors, namely the association (*k*_on_) and dissociation (*k*_off_) rate constants of Equation (1), which give information about the rate of complex formation and its stability

(1) $$K_{D}=\frac{k_{\mathrm{off}}}{k_{\mathrm{on}}}=\frac{1}{K_{A}}$$

Despite being widely used in drug discovery projects, kinetic information alone is insufficient to fully assess the mechanism of binding interactions [18]. In contrast, thermodynamic parameters define the state at equilibrium in which Gibbs free energy (G) is the lowest. Large negative changes in ΔG upon binding define a strong molecular interaction. In fact, the relationship between ΔG and the equilibrium constant (*K*_D_) is mathematically described by the Gibbs equation Equation (2). ΔG can be further decomposed into its enthalpy (ΔH) and entropy (ΔS) components

(2) $$∆G^{o}=\mathrm{RTln}K_{D}=∆H^{o}-T∆S^{o}$$

In Ab–Ag interactions, binding enthalpy ΔH is associated mainly with the strength and specificity of the interaction contacts. These are usually related to hydrogen bonds and electrostatic interactions, although solvent reorganization and conformational change might also have an influence on ΔH. On the other hand, binding entropy (ΔS) reflects the degree of order and disorder of the system and is often linked to the behaviour of surface-bound water molecules, as well as changes in the conformational flexibility of the binding partners. Finally, the temperature dependence of ΔH and ΔS can be defined by changes in the systemic heat capacity (ΔC_p_), as comprised in the van’t Hoff equation Equation (3), where the association constant (*K*_A_) is the inverse of *K*_D_

(3) $$\ln K_{A}=\frac{∆H^{o}T^{o}}{\mathrm{RT}}+\frac{∆S^{o}T^{o}}{R}+\frac{∆C_{p}}{R}\left[\left(\frac{T-{\;T}^{o}}{T}\right)-\ln\left(\frac{T}{T^{o}}\right)\right]$$

Thus, a quantitative assessment of Ab–Ag interactions requires the determination of changes in all thermodynamic parameters, including ΔG, ΔH, ΔS and ΔC_p_. The heat effects (ΔH) of an interaction can be directly measured using isothermal titration calorimetry. However, providing that immobilisation of the protein does not affect its bioactivity, kinetic and thermodynamic parameters can be also calculated from the same experimental dataset using a biosensor technique such as surface plasmon resonance (SPR). Compared to calorimetry measurements, the SPR analysis comes at a much lower time and protein consumption cost.

Our group recently reported the discovery of nanobodies against epidermal growth factor (EGF) [19], a key oncogenic protein involved in the progression of epidermal-based tumours, such as those of the lung, skin and colon, among others [20]. In the present study, we have leveraged the potential of SPR for accurately measuring non-covalent interactions at different temperatures and analyte concentrations to study the kinetics and thermodynamics of two of our leading anti-EGF targeted nanobodies (Nb1 and Nb6). Our analysis thus provides a valuable case study of the distinct molecular forces that drive Ag-Ab binding events, with a special emphasis on the behaviour of single-chain Ab or nanobodies.

## 2. Results

**Figure 1:**
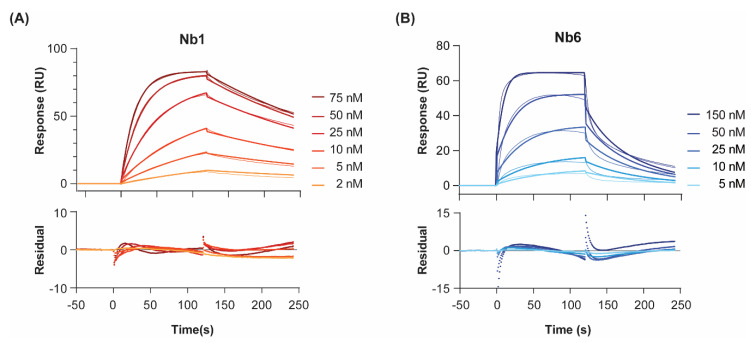
Binding kinetics of Nb1 (**A**) and Nb6 (**B**) to EGF at 25 °C. Nanobodies were injected at increasing concentrations over immobilised EGF, and the association and dissociation phases of all injections were fitted to a 1:1 Langmuir binding model. Two different surface densities were used for *k*_on_, *k*_off_ and *K*_D_ determination, with the corresponding background responses subtracted. Double-referenced sensorgrams for the highest density channel are shown here. Experiments were performed in duplicates. Residual errors from the fits are shown in the bottom panels.

To probe the interactions of Nb1 and Nb6 with their target antigen, we recombinantly expressed human EGF [21] and immobilised it by amine coupling on a CM5 sensor chip. Although different ligand orientations might occur, the three-dimensional dextran hydrogel layer of the chip minimises steric occlusion of the protein. Furthermore, EGF has been shown to maintain its bioactivity intact in amine-coupling immobilisation experiments on SPR chips [22]. Low immobilisation levels of ligand were prioritised (50 and 100 RU, on two different channels) to avoid mass transfer limitations and re-binding artefacts, as well as unspecific non 1:1 interactions [23]. When injected at increasing concentrations, both nanobodies produced, as expected, concentration-dependent SPR sensorgrams and signal saturation at the highest concentrations (Figure 1). We calculated the equilibrium dissociation constants (*K*_D_) at 25 °C, by either (I) plotting the level of steady-state binding for each concentration (i.e., equilibrium fitting, Figure S1); or (II) fitting the association and dissociation signals separately, as a function of time, to a given interaction model (i.e., kinetic fitting, Figure 1).

For both nanobodies binding to EGF, a 1:1 Langmuir interaction model had been previously confirmed by isothermal calorimetry [19]. In agreement, a 1:1 kinetic model provided a close fit to the experimental data, as shown by the low chi-square values (χ^2^ = 0.8–2.9). Regarding the association step, both nanobodies showed fast on-rates (6 and 8 × 10^5^ M^−1^ s^−1^ for Nb1 and Nb6, respectively). On the other hand, the dissociation phase was significantly slower for Nb1. In general, slow binders such as Nb1 reach the SPR steady state (i.e., the timepoint at which the net rate of complex formation is zero) only after very long injection times. In the case of Nb1, it took 300 s to reach 90% of equilibrium at a concentration C equal to its *K*_D_ (6 nM). Since experimental injection times in SPR are typically shorter (in our case, 120 s), more reliable *K*_D_ values are measured by kinetic fitting, which is independent of steady-state levels. It should be noted that these *K*_D_ values are, to some extent, lower than those we previously reported using a different biosensor method, namely surface acoustic wave (SAW) [19]. However, in the SAW technique, the ligand EGF was immobilised at higher densities using a different surface chemistry (alkanethiol monolayer, versus the three-dimensional dextran layer used here for SPR), which could result in mass-transport-limited kinetics, especially for the fastest binders. These differences might explain why, while the affinity (*K*_D_) constants are comparable, SAW-derived on- and off-rates were about an order of magnitude slower than those calculated here by SPR.

**Figure 2:**
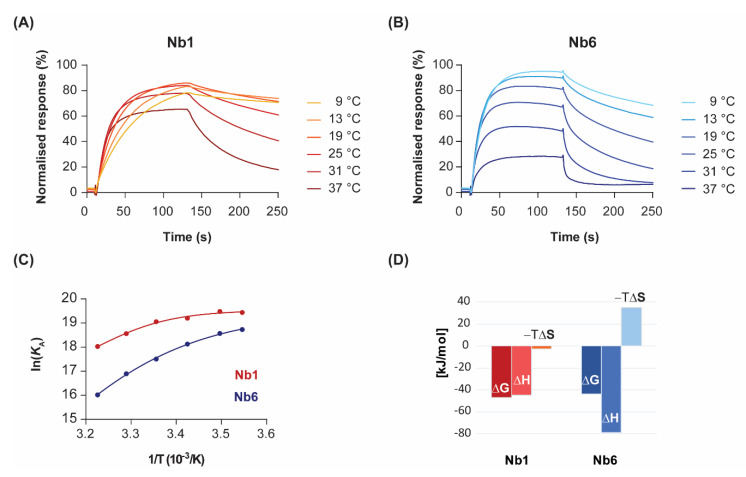
Temperature dependence of binding kinetics and thermodynamic characterisation. Nb1 (**A**) and Nb6 (**B**) were injected over immobilised EGF at the indicated temperatures. For clarity, only sensorgrams of a single analyte concentration (70 nM) are shown. (**C**) Van’t Hoff plots representing the variation of the equilibrium association constants (*K*_A_) with the inverse of temperature. Data points were fitted to a second-order polynomial equation from which ΔG, ΔH, ΔS and ΔC_p_ values were determined, following Equation (3). (**D**) Enthalpy (ΔH) and entropy (−TΔS) contributions to the free energy of binding (ΔG) for each Nb at 25 °C.

To analyse the variation of kinetics with temperature in more detail, we performed SPR experiments at temperatures ranging from 9 to 37 °C (Figure 2). Binding dissociation followed first-order kinetics at all temperatures, as shown by the nearly linear logarithmic plots of the dissociation phases (Figure S2). A 1:1 binding model was applied to extract *k*_on_ and *k*_off_ kinetic constants for each analyte concentration and temperature. The resulting fits, plotted as coloured lines for a single Nb concentration (70 nM) over a range of temperatures (Figure 2A,B), closely described the experimental data. As expected, the kinetics slowed dramatically for both Nbs when the temperature was decreased from room temperature to 9 °C. In contrast, faster association and dissociation sensorgrams were observed with heating to human physiological temperature (37 °C). This effect was more pronounced for the dissociation rate, which translated into an overall drop in the half-life of the complex at the highest temperatures.

By representing the change in the equilibrium association constant (*K*_A_) as a function of temperature, in what is known as the van’t Hoff plot Equation (3), one can gain mathematical access to the thermodynamics of the interaction. In the absence of heat capacity changes, van’t Hoff plots should be linear and thus discriminate between two terms: an enthalpic contribution that is linear in the inverse of temperature (1/T), and a temperature-independent entropic contribution. However, significant curvature in the van’t Hoff plots was observed for both nanobodies at temperatures ranging from 9 to 37 °C (Figure 2C). Although there is always a risk of model overfitting to non-ideal SPR data, the residual values of our global kinetic fitting are very small (see Figure 1, bottom panel). Thus, the lack of linearity in the van’t Hoff plots reveals instead non-zero heat capacity changes (ΔC_p_) for the interactions. The physical reason for the temperature dependence of ΔH is attributed to changes in solvent-accessible surface areas that are buried in the binding process [24]. In particular, the convex shape of the van’t Hoff plots is indicative of negative ΔC_p_, which might result from the burial of hydrophobic protein patches upon complex formation.

Non-linear regression using Taylor’s polynomials allowed the determination of the main thermodynamic parameters for each nanobody (Figure 2D and Table 1). The relative energetic contributions (ΔH and ΔS) are in good agreement with calorimetry data [19]. The binding of Nb1 to EGF was driven by both entropy and enthalpy components, although there was a strong temperature-dependence for ΔH. Since the tangent to the curve at any point of the van’t Hoff plot is ΔH at that precise temperature, the decrease in the Nb1 binding enthalpy is especially evident at lower temperatures. In fact, the binding of Nb1 is purely entropy-driven below 10 °C. In a similar fashion, Nb6 showed a convex van’t Hoff plot indicative of a large negative ΔC_p_ value (Table 1). However, in contrast to Nb1, Nb6 paid a significant entropic penalty for binding its antigen, which was largely compensated by a favourable change in ΔH. As shown here, the negative ΔS is a common feature among most antibodies, since they possess a certain degree of flexibility in the CDR loops that form the antigen-binding site [25]. Besides being attributed to a decreased conformational freedom of the complex, the entropic penalty of Nb6 can also be interpreted in terms of binding to a more hydrophilic epitope on EGF than that of Nb1, thus releasing fewer water molecules into the bulk solvent.

According to transition state theory [26], the temperature dependence of *k*_on_ and *k*_off_ allows analysis of the thermodynamics of activation to form the transition state via the Eyring equation

(4) $$\ln\left(\frac{kh}{k_{B}T}\right)=\frac{∆H^{\neq }T^{o}}{\mathrm{RT}}+\frac{∆S^{\neq }T^{o}}{R}+\frac{∆C_{p}^{\neq }}{R}\left[\left(\frac{T-{\;T}^{o}}{T}\right)-\ln\left(\frac{T}{T^{o}}\right)\right]$$

In a reversible binding process, such as the one studied here, the [EGF-Nb]^≠^ transition state is the high energy state along the binding pathway from free species to complex, which is also visited on the dissociation from the complex to the free species. The thermodynamic approach described herein provides additional information to the classical ITC studies, since SPR monitors the physical association and dissociation of the two molecular species, whereas in calorimetric assays the total heat of the interaction—including heat of dilution or mixing—is measured.

**Figure 3:**
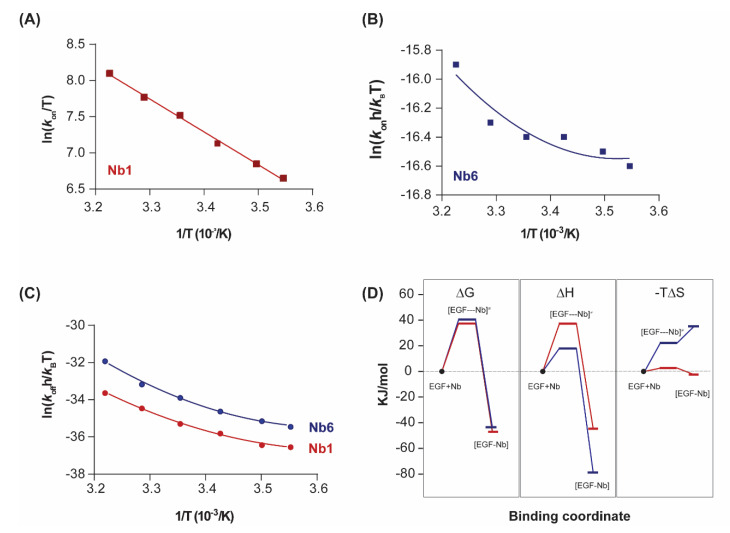
Transition state thermodynamics analysis. (**A**,**B**) Eyring plots representing the variation of the association constant (*k*_on_) with the inverse of temperature for Nb1 and Nb6. Linear fitting of the experimental data points to Equation (4) (ΔC^≠^_p_ = 0) was performed in (**A**), while non-linear fitting was performed in (**B**). (**C**) Eyring plots for the dissociationn step of Nb1 and Nb6. Data were fitted to a second-order polynomial from which the transition state thermodynamic parameters were derived according to Equation (4). (**D**) Binding profile of Nb1 and Nb6 illustrating the transitions in ΔG, ΔH, ΔS at 25 °C. [EGF–Nb]^≠^ represents the transition state.

In its graphical representation, it appears that the Eyring plot for Nb1 association (*k*_on_) is linear but significantly dependent on temperature, corresponding to a large activation enthalpy ΔH^≠^ (Figure 3A). The dissociation Eyring plot (representing *k*_off_) reveals the curvature that is also present in the van’t Hoff plot (Figure 2C), as discussed above. In contrast, for Nb6, both association and dissociation Eyring plots are non-linear (Figure 3B,C). The solution of Eyring’s equation, via Taylor’s series for the non-linear models provides the transition state thermodynamic parameters (denoted by a superscript “≠”).

As shown in Figure 3D, both nanobodies must overcome a significant free energy barrier in order to bind the target. Although enthalpy drives complex formation in both cases, the transition state of Nb6 is characterised by a decrease in the association entropy, which indicates that a reduction in conformational flexibility and structural reordering in the interface is required for binding. This behaviour is in striking contrast to typical protein–protein interactions, in which the entropic changes are typically favourable and thus contribute to an increased affinity [27].

## 3. Discussion

From a biophysical point of view, the analysis of Ab–Ag interactions is often limited to the determination of their binding affinity values. These are sometimes complemented with predictive computational models or high-resolution crystallographic data of the complex. However, the roles of kinetics and thermodynamics in Ab–Ag molecular recognition are frequently overlooked, and this is especially the case for the newer types of antibody formats such as single-chain Abs or nanobodies. However, drawing an analogy with the rational design of small-molecule drugs, it is widely acknowledged that a more detailed notion of the molecular forces driving the interaction is essential to fully understand the binding mechanism of the drug and thus be able to implement rational changes to improve their biological efficacy [28].

Here, we have interrogated the kinetics and thermodynamics of two anti-EGF nanobodies by means of surface plasmon resonance (SPR), a technique that is widely implemented in research laboratories to determine binding affinities. This approach combines high sensitivity and throughput (it can be readily automated for 96- or 384-microwell formats) with low sample consumption and no need of labels for the ligand or the analyte, thus allowing the kinetic and thermodynamic profiling of a large number of preclinical Ab candidates.

Our results show that, for both nanobodies, the formation of the EGF-Ab complex is characterised by negative changes in enthalpy ΔC and heat capacity ΔC_p_ (Figure 2D and Table 1), which are caused by the formation of specific contacts between the two molecules, such as hydrogen bonds and electrostatic interactions, and might be accompanied by the burial of hydrophobic surface patches (negative ΔC_p_). Furthermore, in contrast to Nb6, binding entropy is favourable for Nb1, which, together with its extended on-target residence time, might support its development for potential clinical use.

A key differential feature is the much higher entropy penalty of Nb6 in comparison with Nb1, a difference that already arises in the association step towards the transition state (ΔS^≠^), and which is likely to derive from conformational adjustments of the hypervariable loops at the nanobody binding interface (Figure 3C). The impact of these distinct energetic profiles on the kinetic behaviour of each nanobody (fast off-rate *k*_off_ for Nb6, slow for Nb1) remains an open question. A plausible explanation for this difference, derived from the Eyring analysis of the Nb1 transition state, is that the large dissociation activation enthalpy (ΔH^≠^ = 82.4 kJ/mol) acts as an energetic barrier, preventing the unbinding of Nb1 from the complex once it has formed. Further investigations are nevertheless required.

## 4. Materials and Methods

Recombinant human EGF (MW = 6.2 kDa) was expressed as previously described [21] and was immobilised by amine coupling onto a CM5 sensor chip (series S) using the Biacore T200 (Cytiva, formerly GE Healthcare Life Sciences, Marlborough, MA, USA) at 25 °C. Three different flow cells were independently activated using the regular ratio 1:1 of a 0.4 M 1-ethyl-3-(3-dimethylaminopropyl)-carbodiimide (EDC) and a 0.1 M *N*-hydroxysuccinimide (NHS) for 7 min at 15 µL/min and phosphate buffer saline (PBS) as running buffer. The ligand (EGF) was then injected at 1.87 µM in 10 mM acetate buffer pH 4 in channel 2 and 4 in order to achieve 100 and 50 RU, respectively. Considering the size of the SPR chip (0.5 × 2.9 mm = 1.45 mm^2^), the channel surface is 0.36 mm^2^. Since 1 RU ≈ 1 pg/mm^2^, approximately [29], this results in ≈138 pg of EGF in our low-density channel, while for the high-density channel, ≈275 pg of EGF were immobilised (these numbers compare well with the reported EGF concentration in biological media—human serum EGF is 300–800 pg/mL [30]). The reference channel (flow cell 1) was treated in the same way but with no ligand (EGF) injected. Finally, channels 1, 2 and 4 were blocked using ethanolamine 1 M pH 8.5 for 7 min at 15 µL/min. Channel 3 was left unmodified. Nanobodies were freshly expressed as previously described [19] and used within 1 month of storage at 4 °C, to avoid aggregation artifacts. For affinity, kinetics and thermodynamics experiments, Tween-20 (0.05%) was added to the PBS running buffer.

Kinetic and affinity experiments were performed at 25 °C using a multicycle method, consisting of short regeneration periods (30 s at 30 µL/min of 50 mM HCl) after each analyte injection. Nanobodies were serially diluted at a range of concentrations (75 to 5 nM for Nb1, and 100 to 10 nM, for Nb6) and were injected as analytes for 120 s (followed by 120 s of dissociation) at 30 µL/min. Both high- and low-density channels were combined for global kinetic fittings (see Supporting Data 1 to 3). Thermodynamic studies were performed at a range of temperatures—9, 13, 19, 25, 31, and 37 °C—on the ligand high-density channel. All experiments were performed in duplicate. The resulting sensorgrams were double referenced and fitted to 1:1 Langmuir binding model using Biacore T200 Evaluation Software version 3.1.

Determination of the kinetic and thermodynamic parameters was performed as described below, by fitting the data to Equations (3) and (4) using GraphPad Prism 8 software (see Supporting Data 4 to 7). In Equations (1)–(4), *K*_D_ is the affinity constant (also equilibrium dissociation constant), *K*_A_ is the equilibrium association constant, *k*_on_ and *k*_off_ are the kinetic association and dissociation constants, respectively. ΔG^o^, ΔH^o^, ΔS^o^ and ΔC^o^_p_ are the Gibs free energy, enthalpy, entropy and heat capacity changes under standard conditions (the superscript “^o^” denotes the standard state: 1 atm and 298.15 K; while “≠” refers to the transition state). In Eyring’s equation Equation (4), *k*_B_ is Boltzmann’s constant (1.381 × 10^−23^ J K^−1^) and h is Plank’s constant (6.626 × 10^−34^ J s).

## 5. Conclusions

Our results demonstrate that a comprehensive Ab–Ag interaction analysis, based on thermodynamic and kinetic parameters, can be obtained in a straightforward manner from an SPR biosensor setup. This methodology will prove to be valuable for identifying the underlying structural, thermodynamic and kinetic clues employed by nature-evolved antibodies to recognise their cognate antigens. In addition, since mAb and other Ab formats have currently become one of the most important classes of pharmaceutical agents, we believe that this type of mechanistic analysis will enable researchers to rationally design and engineer improved antibodies with higher affinity, specificity and biochemical stability.

## Figures and Tables

**Table 1:** Summary of all kinetic and thermodynamic constants.

Id	*k*_on_	*k*_off_	*K*_D_^a^	*K*_D_^b^	ΔG^o^	ΔH^o^	−TΔS^o^	ΔC^o^_p_
	(M^−1^ s^−1^)	(s^−1^)	(nM)	(nM)	(kJ mol^−1^)	(kJ mol^−1^)	(kJ mol^−1^)	(kJ K^−1^ mol^−1^)
**Nb1-EGF**	6.6 × 10^5^ ± 5 × 10^3^	3.8 × 10^−3^ ± 2 × 10^−5^	5.8 ± 0.9	16.4 ± 1.2	−47.0 ± 2.2	−44.6 ± 2.4	−2.5 ± 1.9	−2.9 ± 0.5
**Nb6-EGF**	8.8 × 10^5^ ± 3 × 10^4^	2.2 × 10^−2^ ± 8 × 10^−4^	24.3 ± 4.1	30.3 ± 5.3	−43.5 ± 1.4	−78.7 ± 1.4	35.2 ± 1.4	−3.2 ± 0.3

^a^ by means of kinetic fitting; ^b^ steady-state or equilibrium fitting.

## Supplementary Materials

The following are available online at https://www.mdpi.com/1424-8247/13/6/134/s1, Figure S1: SPR equilibrium responses of Nb1 (**A**) and Nb6 (**B**) at a range of concentrations. KD values resulting from non-linear curve fitting to a 1:1 Langmuir model are reported in Table 1 (main text).; Figure S2: Semilogarithmic plots of the dissociation phases for Nb1 (**A**) and Nb6 (**B**) at 50 nM over a range of temperatures, indicating that dissociation largely follows first-order kinetics at each temperature (note the exception for Nb6 at 37 °C, with a very fast dissociation that deviates from linearity).
