# Supplementary material for: Probing the Kinetic and Thermodynamic Fingerprints of Anti-EGF Nanobodies by Surface Plasmon Resonance
Source: Pharmaceuticals (Basel). 2020 Jun 26;13(6):134. doi: 10.3390/ph13060134 (PMC7344977; doi:10.3390/ph13060134)
Supplement: Supplementary file 1 [file pharmaceuticals-13-00134-s001.pdf]

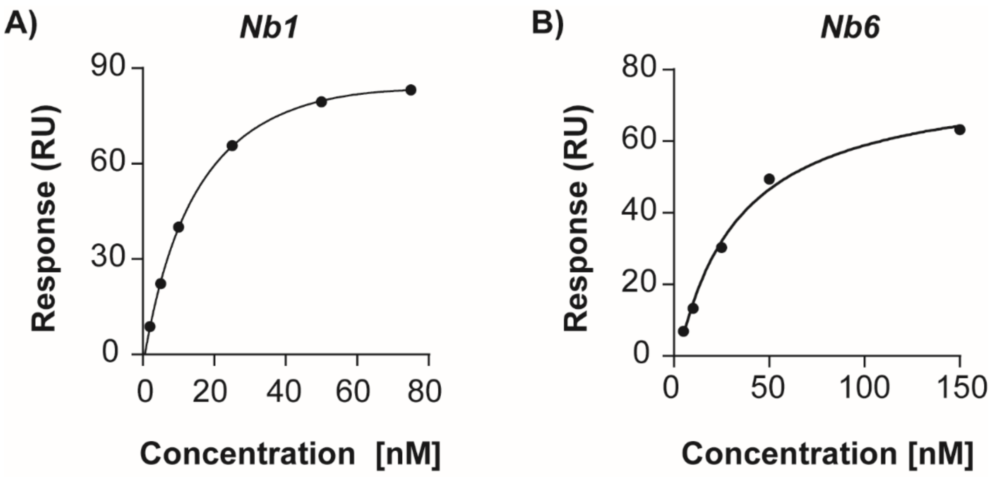

Supporting Figure S1. SPR equilibrium responses of Nb1 (A) and Nb6 (B) at a range of concentrations. K_D values resulting from non-linear curve fitting to a 1:1 Langmuir model are reported in Table 1 (main text).

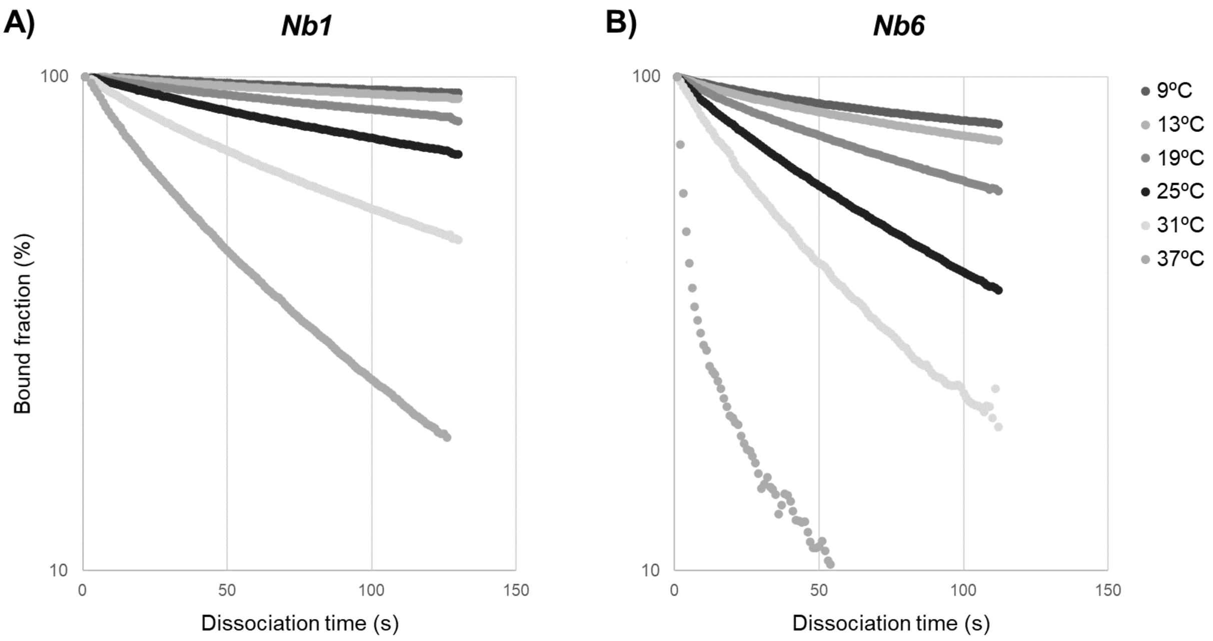

Supporting Figure S2. Semilogarithmic plots of the dissociation phases for Nb1 (A) and Nb6 (B) at 50 nM over a range of temperatures, indicating that dissociation largely follows first-order kinetics at each temperature (note the exception for Nb6 at 37ºC, with a very fast dissociation that deviates from linearity).

Supporting Data 1. Global kinetic fittings for Nb1 (left) and Nb6 (right) at 25ºC.

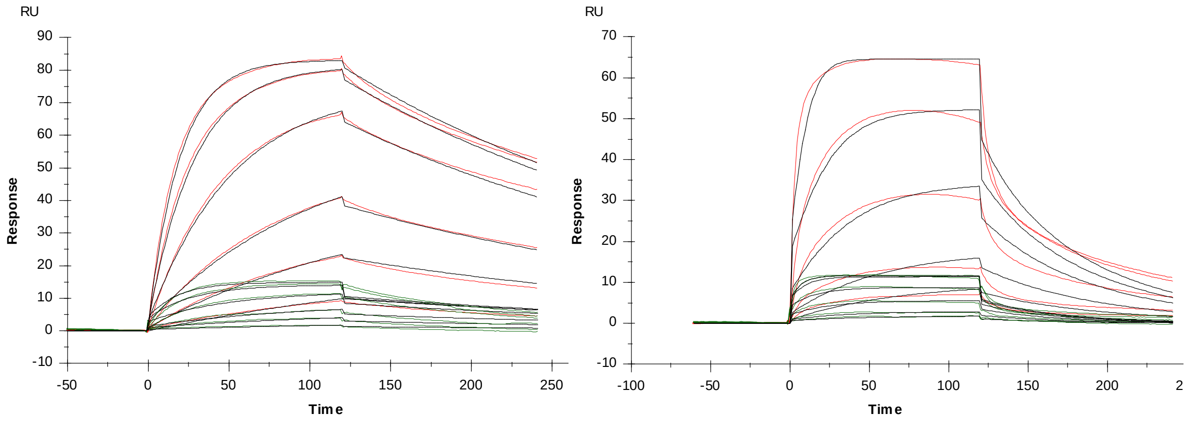

	ka (1/Ms)	SE(ka)	kd (1/s)	SE(kd)	Rmax_1 (RU)	Rmax_2 (RU)	tc	Chi² (RU²)	U-value
**Nb1**	6.64E+05	5.50E+03	3.83E-03	2.50E-05	87.5	11.5	2.51E+08	0.76	1
**Nb6**	8.82E+05	3.10E+04	2.15E-02	7.80E-04	52.7	6.9	2.01E+07	2.28	4

Supporting Data 2. Global equilibrium fittings for Nb1 (left) and Nb6 (right) at 25ºC.

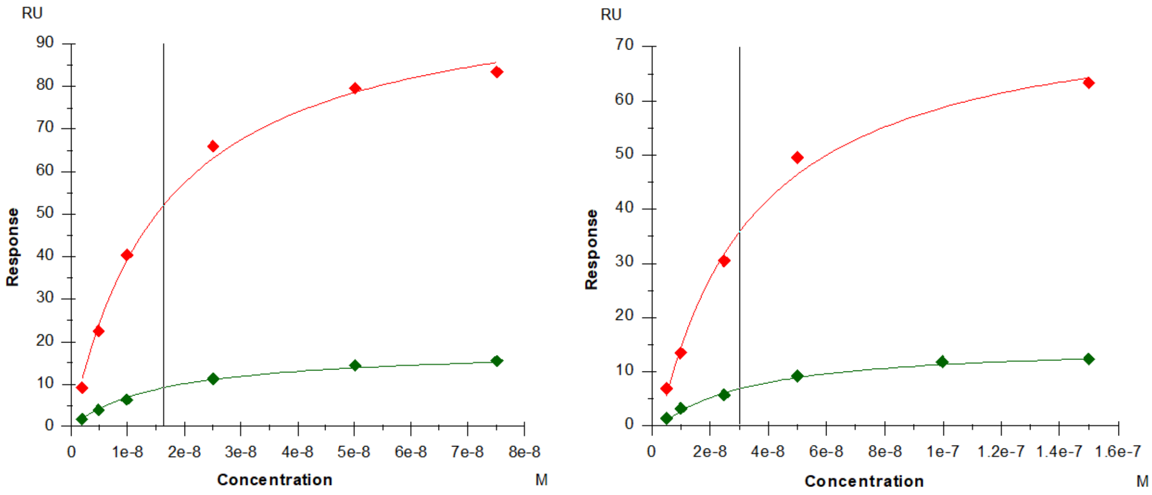

		KD (M)	Rmax (RU)	offset (RU)	Chi² (RU²)
Nb1	Fitting	1.64E-08			2.87
Nb1	Curve 1		104.2	0	
Nb1	Curve 2		18.36	0	
Nb6	Fitting	3.03E-08			2.76
Nb6	Curve 1		85	-6.425	
Nb6	Curve 2		16.5	-1.299	

Supporting Data 3. Global kinetic fittings for Nb1 (left) and Nb6 (right) at 9, 13, 19, 25, 31, and 37ºC.

9ºC

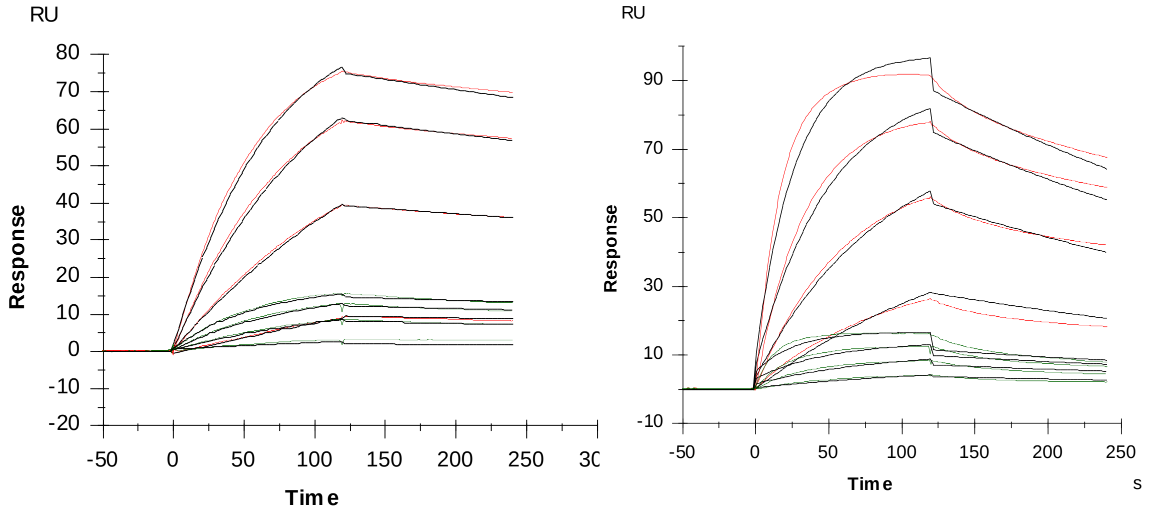

13ºC

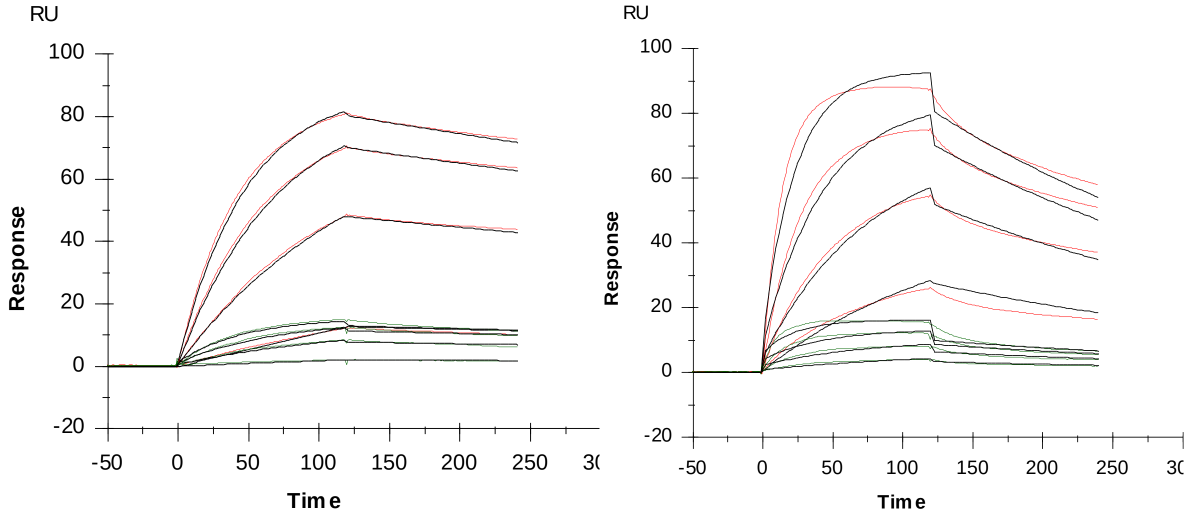

19ºC

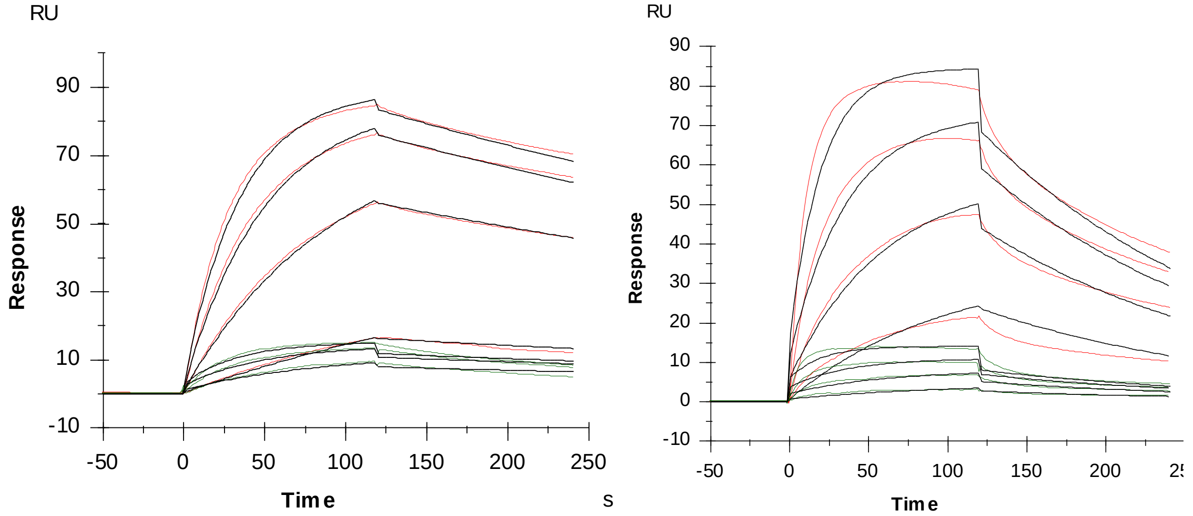

25ºC

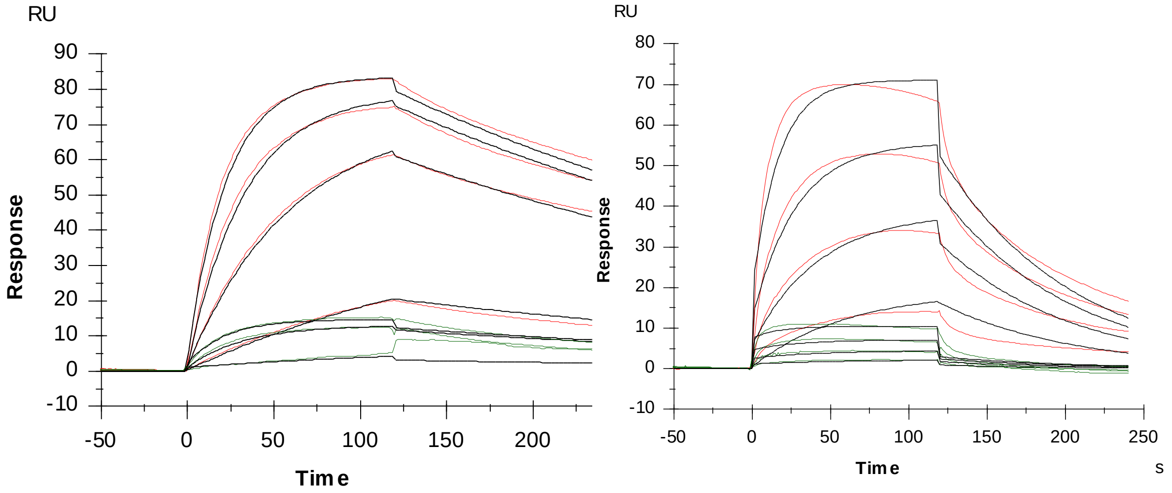

31ºC

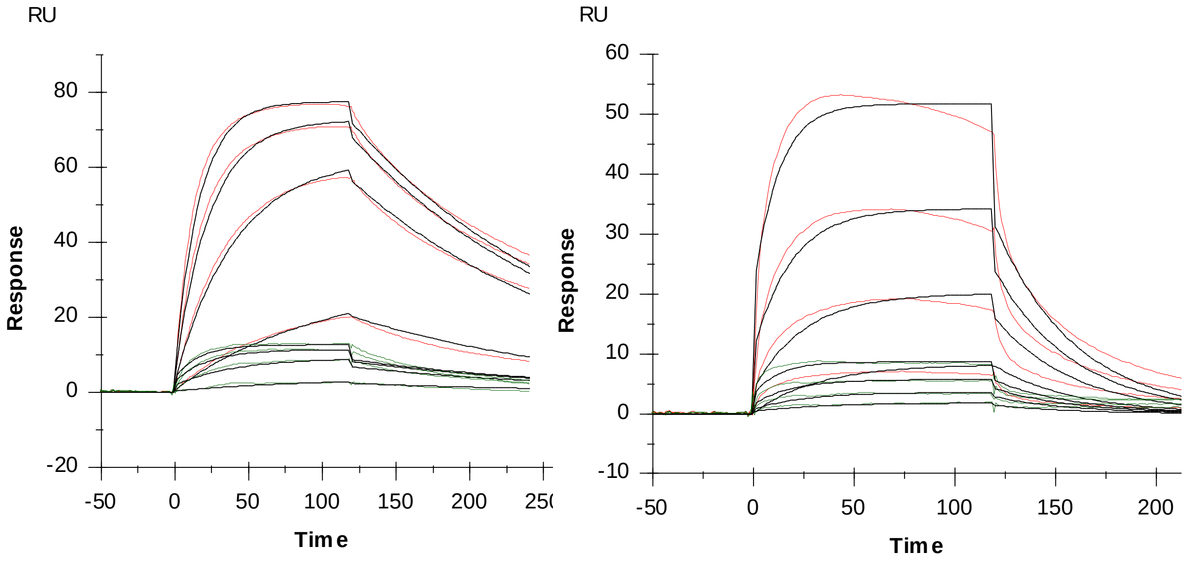

37ºC

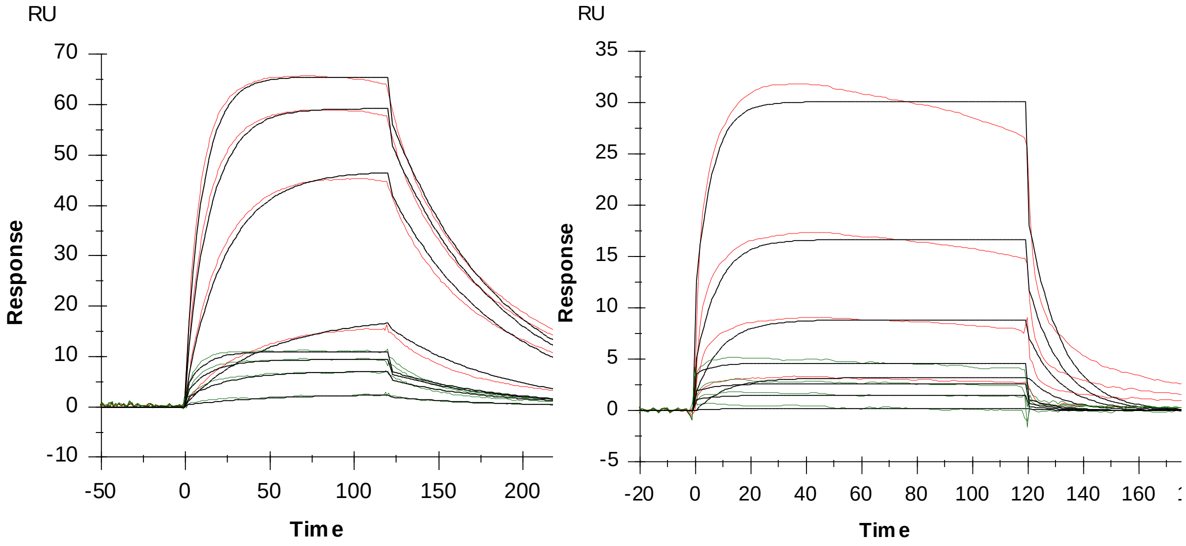

Supporting Data 4. Van't Hoff analysis for Nb1.

								Y-axis			X-axis
Evaluation	Temperature (°C)	Model	kon (1/Ms)	koff (1/s)	**KD (M)**	ln(KD)	Ka (M-1)	ln(Ka)	T (K)	1/T	1/T (x100)
NB3	9	1:1 Binding	2.19E+05	7.89E-04	3.61E-09	-19.439	2.77E+08	19.439	282	0.003546	3.5461
NB3 2	13	1:1 Binding	2.70E+05	9.36E-04	3.46E-09	-19.4817	2.89E+08	19.48171	286	0.003497	3.4965
NB3 3	19	1:1 Binding	3.65E+05	1.67E-03	4.58E-09	-19.2024	2.19E+08	19.20244	292	0.003425	3.4247
NB3 4	25	1:1 Binding	5.49E+05	2.90E-03	5.29E-09	-19.0569	1.89E+08	19.05688	298	0.003356	3.3557
NB3 5	31	1:1 Binding	7.24E+05	6.29E-03	8.70E-09	-18.5604	1.15E+08	18.5604	304	0.003289	3.2895
NB3 6	37	1:1 Binding	1.02E+06	1.51E-02	1.48E-08	-18.0259	6.74E+07	18.02594	310	0.003226	3.2258

Fitting to a second-order polynomial equation:

$$\ln K_{\mathrm{eq}} = a + \frac{b}{T} + \frac{c}{T^2},$$

where

$$\Delta H = -R\left(b + \frac{2c}{T}\right),$$

$$\Delta S = R\left(a - \frac{c}{T^2}\right).$$

Second order polynomial (quadratic)		Results:		
GraphPad	Y=B0 + B1*X +B2*X^2	dH	-44.5594	kJ/mol
		dS	0.008296	kJ/K·mol
B0	-1.75E+02	-TdS	-2.47218	kJ/mol
B1	1.10E+05	dG	-47.0316	kJ/mol
B2	-1.56E+07	dCp	-2.91815	kJ/K·mol
R2	0.99			

Supporting Data 5. Van't Hoff analysis for Nb6.

								Y-axis			X-axis
Evaluation	Temperature (°C)	Model	kon (1/Ms)	koff (1/s)	**KD (M)**	ln(KD)	Ka (M-1)	ln(Ka)	T (K)	1/T (x100)	1/T (x100)
NB3	9	1:1 Binding	3.47E+05	2.55E-03	7.34E-09	-18.7301	1.36E+08	18.73006	282	0.003546	3.5
NB3 2	13	1:1 Binding	3.92E+05	3.38E-03	8.63E-09	-18.5684	1.16E+08	18.56837	286	0.003497	3.5
NB3 3	19	1:1 Binding	4.39E+05	5.88E-03	1.34E-08	-18.128	7.46E+07	18.12801	292	0.003425	3.4
NB3 4	25	1:1 Binding	4.76E+05	1.19E-02	2.49E-08	-17.5068	4.01E+07	17.50679	298	0.003356	3.4
NB3 5	31	1:1 Binding	5.49E+05	2.53E-02	4.60E-08	-16.8938	2.17E+07	16.89376	304	0.003289	3.3
NB3 6	37	1:1 Binding	7.77E+05	8.58E-02	1.10E-07	-16.0201	9.07E+06	16.02006	310	0.003226	3.2

Fitting to a second-order polynomial equation:

$$\ln K_{\text{eq}} = a + \frac{b}{T} + \frac{c}{T^2},$$

where

$$\Delta H = -R\left(b + \frac{2c}{T}\right),$$

$$\Delta S = R\left(a - \frac{c}{T^2}\right).$$

Second order polynomial (quadratic)		Results:		
GraphPad	Y=B0 + B1*X +B2*X^2	dH	-78.6752	kJ/mol
		dS	-0.11798	kJ/K·mol
B0	-204.8	-TdS	35.15683	kJ/mol
B1	123067	dG	-43.5183	kJ/mol
B2	-1.7E+07	dCp	-3.16782	kJ/K·mol
R2	0.991			

Supporting Data 6. Eyring analysis for Nb1.

						Y-axis		Y-axis			X-axis
Evaluation	Temperature (°C)	Model	kon (1/Ms)	koff (1/s)	**KD (M)**	ln(Ka/T)	ln(Kd/T)	ln(kdh/kBT)	T (K)	1/T	1/T (x100)
NB3	9	1:1 Binding	2.19E+05	7.89E-04	3.61E-09	6.65	-12.79	-36.5	282	0.003546	0.35461
NB3 2	13	1:1 Binding	2.70E+05	9.36E-04	3.46E-09	6.85	-12.63	-36.4	286	0.003497	0.34965
NB3 3	19	1:1 Binding	3.65E+05	1.67E-03	4.58E-09	7.13	-12.07	-35.8	292	0.003425	0.342466
NB3 4	25	1:1 Binding	5.49E+05	2.90E-03	5.29E-09	7.52	-11.54	-35.3	298	0.003356	0.33557
NB3 5	31	1:1 Binding	7.24E+05	6.29E-03	8.70E-09	7.77	-10.79	-34.5	304	0.003289	0.328947
NB3 6	37	1:1 Binding	1.02E+06	1.51E-02	1.48E-08	8.10	-9.93	-33.7	310	0.003226	0.322581

Association

Linear Eyring

slope	-4539	dH	37.71909	kJ/mol
Y-intercept	22.72	dS	-0.00864	kJ/K·mol
		-TdS	2.57538	kJ/mol
		dG	40.29447	kJ/mol
		dCp	0	kJ/mol

Dissociation

Non-linear Eyring

Second order polynomial (quadratic)

GraphPad Y=B0 + B1*X +B2*X^2

B0	187.7	dH	82.35037	kJ/mol
B1	-122975	dS	-0.01668	kJ/K·mol
B2	16846716	-TdS	4.969413	kJ/mol
		dG	87.31978	kJ/mol
R2	0.999129	dCp	3.152926	kJ/K·mol

Constants for standard conditions (°):

T	298	K
R	0.00831	kJ/K·mol
kB	1.38E-23	J/K
h	6.63E-34	Js
k	1	

Supporting Data 7. Eyring analysis for Nb6.

							Y-axis		Y-axis			X-axis
Evaluation	Temperature (°C)	Model	kon (1/Ms)	koff (1/s)	**KD (M)**	ln(Ka/T)	ln(kah/kBT)	ln(Kd/T)	ln(kdh/kBT)	T (K)	1/T	1/T (x1000)
NB3	9	1:1 Binding	3.47E+05	2.55E-03	7.34E-09	7.12	-16.6	-11.61	-35.4	282	0.003546	3.5
NB3 2	13	1:1 Binding	3.92E+05	3.38E-03	8.63E-09	7.22	-16.5	-11.34	-35.1	286	0.003497	3.5
NB3 3	19	1:1 Binding	4.39E+05	5.88E-03	1.34E-08	7.32	-16.4	-10.81	-34.6	292	0.003425	3.4
NB3 4	25	1:1 Binding	4.76E+05	1.19E-02	2.49E-08	7.38	-16.4	-10.13	-33.9	298	0.003356	3.4
NB3 5	31	1:1 Binding	5.49E+05	2.53E-02	4.60E-08	7.50	-16.3	-9.39	-33.2	304	0.003289	3.3
NB3 6	37	1:1 Binding	7.77E+05	8.58E-02	1.10E-07	7.83	-15.9	-8.19	-32.0	310	0.003226	3.2

Association

Non-linear Eyring

B0	64.68	dH	18.22634	kJ/mol
B1	-46111	dS	-0.07485	kJ/K·mol
B2	6543737	-TdS	22.30578	kJ/mol
		dG	40.53212	kJ/mol
R2	0.9	dCp	1.224685	kJ/K·mol

Dissociation

Non-linear Eyring

Second order polynomial (quadratic)

GraphPad Y=B0 + B1*X +B2*X^2

B0	247.3788	dH	96.30985	kJ/mol
B1	-156073	dS	0.041198	kJ/K·mol
B2	21527976	-TdS	-12.2769	kJ/mol
		dG	84.03295	kJ/mol
R2	0.996	dCp	4.029041	kJ/K·mol

Constants for standard conditions (°):

T	298	K
R	0.00831	kJ/K·mol
kB	1.38E-23	J/K
h	6.63E-34	Js
k	1	
